# Selected Domains within a Comprehensive Geriatric Assessment in Older Patients with Non-Hodgkin Lymphoma are Highly Associated with Frailty

**DOI:** 10.1007/s44228-022-00005-7

**Published:** 2022-05-11

**Authors:** María del Pilar Gamarra Samaniego, Carmelo J. Blanquicett, Roger V. Araujo Castillo, Julio C. Chavez, Brady Ernesto Beltrán Garate

**Affiliations:** 1 Department of Internal Medicine, Hospital Nacional Edgardo Rebagliati Martins (HNERM)-ESSALUD, Lima, Peru; 2 Department of Malignant Hematology, Moffitt Cancer Center, 12902 Magnolia Drive, Tampa, FL 33612 USA; 3 Dirección de Investigación en Salud–Instituto de Evaluación de Tecnologías de Salud e Investigación (IETSI)-ESSALUD, Lima, Peru; 4 Servicio de Oncología Clínica-Hospital Nacional Edgardo Rebagliati Martins (HNERM)-ESSALUD, Lima, Peru; 5 Instituto de Ciencias Biomedicas, Universidad Ricardo Palma, Lima, Peru

**Keywords:** Comprehensive geriatric assessment, Non-Hodgkin lymphoma, Frailty, Geriatric oncology

## Abstract

**Background:**

The incidence of Non-Hodgkin Lymphoma (NHL) is increasing, particularly among older patients who tend to have worse outcomes and can be predisposed to increased toxicities and less treatment tolerance. Therefore, a thorough pre-treatment assessment is essential. A comprehensive geriatric assessment (CGA) can be used to evaluate the older patient considering chemotherapy and is the preferred evaluation tool. However, a formal CGA is laborious, complex and time-consuming.

**Objectives:**

To characterize older adults with NHL and determine the CGA variables with the greatest association to frailty in order to propose a more simplified assessment.

**Methods:**

We performed a cross-sectional study using data collected from CGAs in NHL patients > 65 years admitted to our oncology service, from September 2015 to August 2017. Our evaluation parameters included: polypharmacy, a screening tool of older people's prescriptions (STOPP), the Lawton scale, Barthel index, Katz index, gait speed, a Timed Up and Go (TUG) test, a Mini-Mental state examination (MMSE), the Yesavage and Gijon scales, a Mini-nutritional assessment (MNA), a Geriatric Syndromes assessment, and a Cumulative Illness Rating Scale-Geriatric (CIRS-G). The formal CGA was comprised of nine domains; frailty was defined as an impairment in > 2 domains. Each parameter was individually compared with frailty, and the results were used to build different multivariate models using logistic regression analyses to obtain the variables with the highest frailty association.

**Results:**

A total of 253 patients were included. Their median age was 75.4 years (range 65–92), and 62.1% had > 1 impaired domain, with 39.9% considered frail. Bivariate analysis showed strong associations with age > 85 and all the geriatric parameters except for STOPP. Our final multivariate analysis resulted in 5 domains (the use of > 5 medications, a Lawton < 7, TUG > 20, Yesavage > 5, and the presence of at least one geriatric syndrome) being significantly associated with frailty and performing similarly to a CGA.

**Conclusion:**

In our population of older NHL patients, an abbreviated evaluation based of only five domains, polypharmacy, TUG, Lawton scale, Yesavage scale and the presence of at least one geriatric syndrome, had similar performance to a formal CGA in determining frailty.

## Introduction

The incidence of non-Hodgkin’s lymphoma (NHL) continues to increase, especially in patients older than 60 years [1]. The most common NHL-subtype is diffuse large B-cell lymphoma (DLBCL) [2]. This overall increased NHL incidence has also been observed in Lima, Peru. For instance, between 1968 and 1970, the incidence was 3.4 and 6 per 100 000 in females and males, respectively, whereas between 1994 and 1997 it increased to 7.5 and 8.3 per 100 000 in females and males, respectively [3]. Beltrán et al. reported that approximately 75% of cases were of the B-cell type and 52% were nodal lymphomas in Peru [4]. DLBCL is not only the most common type of NHL in older patients but also has a poorer prognosis, mainly due to the presence of a greater number of comorbidities and impairments in functional status that are commonly observed among the elderly [5]. These factors could affect the ability of older adults to tolerate treatment.

Thus, pre-treatment evaluation of the geriatric patient is essential in order to determine the best treatment plan for that patient [6]. Different geriatric patient characteristics are strongly associated with treatment outcomes. Examples of this are the associations between frailty, nutritional status and comorbidities with increased mortality, frailty alone and increased toxicity to chemotherapy; impairments in cognition and activities of daily living (ADLs) as predictors of treatment noncompliance; impairments in instrumental ADLs (iADLs) and perioperative complications [7]. Finally, frailty, grip strength, physical activity, nutrition, mobility, independence, depression, impaired ADLs, history of falls and ECOG performance status have been shown to influence the final clinical decision regarding treatment [8]. Unfortunately, ECOG performance status may not be adequate when assessing the older cancer patient’s ability to tolerate treatment. The same limitations associated with using the ECOG performance scale are encountered when using the Karnofsky Performance Status Scale.

The comprehensive geriatric assessment (CGA) is an evaluation method used by geriatricians to describe the multidisciplinary assessment of an older patient. The CGA can be applied to the evaluation of an older patient considering chemotherapy, as it was developed to assess the aforementioned factors that are pertinent to the older adult, including functional status, comorbidities, social interactions and psychological factors [9]. The CGA includes several domains: functional capacity, performance status, comorbidities, polypharmacy, cognition, nutritional status, psychological status and social support [10]. The National Comprehensive Cancer Network (NCCN) and many groups recommend that formal CGAs be employed to assess all patients older than 65 years with a cancer diagnosis, in order to identify health issues that can predispose them to increased adverse outcomes [11, 12]. The CGA has consistently proven to be associated with overall survival in cancer patients, and is considered the gold standard tool, as compared to alternative methods of pre-treatment assessments. A systematic review [13] found 11 high-quality studies that reported the CGA to be associated with overall survival, with even poorer outcomes reported when more domains were affected. In the specific case of NHL, a prospective study, the Fondazione Italiana Linfomi (FIL), reported that patients classified as frail had worse survival outcomes than “fit” patients, despite receiving the same rituximab-based chemotherapy regimen, with a HR of 2.37 (IC 95% 1.48–3.78) [14].

Despite its proven value, the CGA is complex, timeconsuming, and constitutes a demanding process that may be impractical to apply to all patients in a busy oncology practice. More simple screening tools that can be quickly administered have been developed to identify patients who may require a more complete assessment [15]. While these tools were shown to have utility, they can miss several cases of older adults with cancer who might be rated as functionally normal by that measure but have deficits identified on a CGA. Moreover, the best clinical tools have a negative predictive value of 60, meaning that out of 5 patients not considered frail through these screening tools, 2 would have been classified as frail using the more thorough CGA [16]. Given the relevance of the CGA in the management of older patients with cancer, we aimed to evaluate whether this tool is applicable in Peru and whether specific domains have higher association with frailty in our patient population, thus simplifying the CGA by the identification of the more relevant domains or variables that could be predictive of frailty.

The objective of our study was to determine the demographic and clinical characteristics of patients with NHL, age 65 and older, managed in a tertiary hospital, on whom CGA evaluations were performed to further determine which of the formal CGA variables would have the greatest predictive value of frailty, so as to propose an abbreviated assessment tool.

## Methods

In this cross-sectional, retrospective study, we evaluated geriatric patients aged 65 and older, with a proven diagnosis of NHL, who were treated in the Oncology Service at the Hospital Nacional Edgardo Rebagliati Martins (HNERM) between September 2015 and August 2017. Patients were excluded if: there was no confirmed NHL diagnosis, if geriatric evaluations were incomplete, or if they received therapy elsewhere outside the Oncology Service; patients with transformed follicular lymphoma (TFL) were also excluded. A census of the entire study population (eligible and excluded patients who did not meet inclusion criteria) was created during the study period.

Participants were identified by the evaluations provided by the HNERM Geriatric Unit in the Oncology Service during the aforementioned study period. The geriatric unit gathers demographic data (gender, age, race, origin), clinical data (diagnosis, vital signs, weight, height, body mass index [BMI], complete blood counts and lactate dehydrogenase [LDH] levels), several performance status and geriatric assessments including: Vulnerable Elder Survey 13 (VES13), 8-min questionnaire for the elderly, Geriatric 8 (G8), Eastern Cooperative Oncology Group (ECOG) functional scale, polypharmacy, screening tool of older person’s prescriptions (STOPP) to detect inappropriate medications, Lawton scale, Barthel scale, Katz index, gait speed, timed Up and Go (TUG), Mini-Mental Status Exam (MMSE), Yesavage scale, Gijón scale, Mini-nutritional Assessment scale (MNA), presence of geriatric syndromes (delirium, dementia, incontinence, depression, falls history, polypharmacy, pressure ulcers, immobility, sensory deficit, osteoporosis), Cumulative Illness Rating Scale-Geriatric (CIRS-G) and CIRS-G based severity index. Altogether, these data were utilized in order to produce a formal CGA that included 9 domains: IADLs, polypharmacy defined as > 5 drugs, IADLs using the Lawton scale, functional status indirectly measured using the TUG, cognitive status using the MMSE, psychiatric status using the Yesvage scale, social status using the Gijon scale, nutritional status using a MNA, the presence of geriatric syndromes, and comorbidities using the CIRS-G. Frailty was considered to be present if two or more domains were affected.

The geriatric unit records were reviewed, and those with complete information with respect to the CGA were recorded in an Excel file. Categorical variables were built using this database and considered normal or abnormal using standard definitions which have been previously described. These are noted in Table 1. The database was proofread to detect errors, duplications or omissions. Finally, the database was de-identified for further analysis of tools that were not included in these analyses such as the VES13, G8 and ECOG screening tools. Categorical variables were described using frequencies, percentages and confidence intervals (CI) set at 95%. Continuous variables were described using medians, means, standard deviations, maximum and minimum values. In order to determine the association between the CGA and the different variables obtained (including each domain within the CGA) we utilized chi-square tests. The strength of association was quantified using odds ratios (OR). Variables with significant associations and which did not have co-linearity were used to build multivariate models. Co-linearity was assessed using simple linear correlation analyses, and the variable with the weakest association was eliminated. The multivariate analysis was performed using the ORs modeled by the logistic multiple regression analyses. The model was performed by adding the variables in descending order, starting with the variable with the strongest association. A *p* value < 0.05 was considered significant, using the statistics program STATA, version 12.0 (College Station, TX).

**Table 1:** General characteristics and geriatric scales included in the comprehensive geriatric evaluation (CGA)

Variables	*N* = 253 (%col)	95% CI (except^a^)
Age		
Mean	75.61	6.68^a^
Median	75	65–92^b^
65–74 years	45.02%	38.8–51.4%
75–84 years	42.63%	36.4–49.0%
> 85 years	12.35%	8.6–17.1%
Gender		
Female	53.36%	47.0–59.6%
Education		
< 4 years	16.4%	11.1–22.9%
4–7 years	24.2%	17.9–31.5%
> 7 years	59.4%	51.5–67.0%
Polypharmacy		
> 5 drugs	10.28%	6.8–14.7%
STOPP		
Restricted	10.50%	6.9–15.1%
Lawton Scale		
Mean	6.04	2.40^a^
Median	7	0–8^b^
< 7	36.51%	30.6–42.8%
Barthel Scale		
Mean	81.46	25.56^a^
Median	95	0–100^b^
≤ 60	21.83%	16.9–27.4%
Katz index		
Mean	2.34	2.44^a^
Mediann	1	0–6^b^
Categories 2–6	48.00%	41.7–54.4%
Walking speed		
Mean	0.98	1.46^a^
Median	0.67	0.07–15^b^
< 0.8	61.05%	53.7–68.0%
Time up and go		
Mean	15.96	7.56^a^
Median	15.1	3.3–67^b^
> 20	15.76%	10.8–21.8%
Mini-mental (MMSE)		
Mean	25.15	4.80^a^
Median	27	4–30^b^
< 23	24.70%	19.5–30.6%
Yesavage scale		
Mean	4.50	2.31^a^
Median	4	0–13^b^
> 5	27.71%	22.3–33.7%
Gijón scale		
Mean	5.78	2.34^a^
Median	5	2–17^b^
≥ 10	4.78%	2.5–8.2%
Body mass index (BMI)		
Mean	24.52	3.95^a^
Median	24.2	13.2–37.5^b^
< 19	4.96%	2.6–8.5%
Mini-nutritional		
Mean	20.80	4.10^a^
Median	22	0–30^b^
< 17	12.96%	9.0–17.8%
17–23	59.51%	53.1–65.7%
> 23	27.53%	22.1–33.6%
# Geriatric syndromes		
Mean	1.03	0.98^a^
Median	1	0–4^b^
At least one	66.40%	60.2–72.2%
CIRS—G		
Mean	3.93	4.30^a^
Median	2	0–9^b^
> 2	52.71%	45.6–59.7%
# Affected domains of the comprehensive geriatric assessment		
Mean	2.36	1.76^a^
Median	2	0–7^b^
> 1	62.06%	55.8–68.1%
> 2	39.92%	33.8–46.2%

^a^Standard Deviation

^b^Range Minimum–Maximum

This study had minimal risks to patients because it was a retrospective review of clinical data. The forms were routinely completed in the Geriatric Unit, thus additional procedures were not needed for our study. Consequently, specific authorization from the Institution was not required. The information collected was de-identified and coded by the principal investigator (PI) who maintained appropriate confidentiality. The study was reviewed and approved by the Institutional Review Board and Ethics Committee of the HNERM.

## Results

A total of 269 forms were collected from individual patients. Of these, 253 were 65 years or older, and had a proven diagnosis of NHL. The median age was 75.4 (range 65–92) years with 12.4% of patients being older than 85 years, as shown in Table 1. Fifty-three percent were female and 59.4% of patients had more than 7 years of education. The different geriatric evaluations (as well as the percentages of abnormal results) are described in Table 1. Each patient had a mean of 2.36 (SD + 1.76) domains affected within a CGA. Among all subjects evaluated, 62.1% and 39.9% had at least 1 and 2 or greater domains affected, respectively; therefore, they were categorized as frail.

Of interest, different scales capturing similar measures displayed variation. The Barthel scale resulted in 21.8% of patients deemed as having total or severe dependence, whereas the Katz scale resulted in a nearly-double proportion of patients having total dependence. Although both scales aim to capture similar measures, a numeric correlation was not found. The Lawton index, which measures instrumental activities, had intermediate results between the 2 aforementioned scales. Another similar situation occurred with the gait speed and the TUG, which were abnormal in 15.8% and 63.2% of patients, respectively, despite both tests being similar in what they represent.

Malnutrition or BMI ≤ 19 was present in 5% of the study population, and 13% were found to be malnourished by the mini-nutritional assessment (MNA). It is important to point out that these assessments were performed pre-treatment and the effect of chemotherapy on the nutritional status was not considered.

In our study, the Yesavage scale revealed higher rates of depression (28%) than previously reported [17]. Cognitive impairment, as assessed by MMSA, was similar to that from previous study in cancer patients [18, 19]. Two-thirds of our patients had at least one geriatric syndrome (excluding dementia, delirium, depression and polypharmacy) in this study. Similarly, over half of the subjects had a CIRS-G score ≥ 2, reflecting the high prevalence of comorbidities.

Excluding the rapid tests, such as the VES13 and G8, the different scales which were part of the CGA were strongly associated with frailty. The presence of a geriatric syndrome had the strongest association with frailty. This is highlighted by a low prevalence of frailty (7.1%) in the absence of a geriatric syndrome, endowing the latter with a high negative predictive value. The only scale without a significant association was the STOPP, as this tool is designed to measure potentially inappropriate medications in older adults rather than detecting frailty. On the other hand, factors such as polypharmacy, Lawton scale, Barthel scale, MNA and TUG had a high positive predictive value (PPV), given their high abnormality rate (in more than 80% of cases); these are suggested to be excellent markers of frailty.

### Bivariate and Multivariate Analysis

The bivariate analysis found variables with some or high degree of linear correlation, (Table 2). The two scales that measure ADLs (Barthel and Katz) had a low degree of correlation (*r* < 0.05) between them, despite measuring similar items. Interestingly, these scales were associated with other measures, for instance, the Barthel scale had strong co-linearity with the Lawton scale, despite the latter measuring instrumental ADLs. Moreover, the Katz scale was associated with the TUG, despite being a significantly different measure. It is also of interest to note that the Katz scale did not have correlation with other ADL measurements or IADLs, despite its association with the TUG. One possible explanation is that some of the scales preferentially measure physical activity -such as the Katz scale, hence its correlation to the TUG. Alternatively, they may provide a more effective measure of the cognitive aspect of the instrumental activities (such as the Lawton scale and the correlation with IADL indices). Unsurprisingly, the CIRS-G was associated with the presence of geriatric syndromes, as several of these variables already comprise part of the CIRS-G tool. When there were 2 co-linear variables, the one with the strongest association was chosen for the multivariate model. It is a general assumption that the effect of the variable with the weaker association affects the strength of the variable with the strongest association. Thus, the final model excluded Barthel, Katz and the CIRS-G.

**Table 2:** Bivariate analysis using frailty as a dependent variable (as defined per the study: with 2 or more comprehensive geriatric assessment domains affected)

Variables	CGA > 2/9 *N* = 101 (% column)	CGA ≤ 2/9 *N* = 152 (% column)	Prevalence ratios (95% CI)	*p* value (X^2^ except^a^)
Age				
65–74 year	36 (31.9%)	77 (68.1%)	–	–
75—84 years	44 (41.1%)	63 (58.9%)	1.29 (0.91:1.84)	0.153
> 85	21 (67.7%)	10 (32.3%)	2.13 (1.48:3.06)	< 0.001
Polypharmacy				
≤ 5 drugs	79 (34.8%)	148 (65.2%)	–	–
> 5 drugs	22 (84.6%)	4 (15.4%)	2.43 (1.91:3.10)	< 0.001
Lawton scale				
≥ 7	25 (15.6%)	135 (84.4%)	–	–
< 7	75 (81.5%)	17 (18.5%)	5.22 (3.59:7.58)	< 0.001
Barthel scale				
> 60	56 (28.4%)	141 (71.6%)	–	–
≤ 60	44 (80.0%)	11 (20.0%)	2.81 (2.17:3.64)	< 0.001
Katz index				
Categories 0–1	32 (24.6%)	98 (75.4%)	–	–
Categories 2–6	67 (55.8%)	53 (44.2%)	2.27 (1.61:3.19)	< 0.001
Walking speed				
≥ 0.8	10 (13.5%)	64 (86.5%)	–	–
< 0.8	44 (37.9%)	72 (62.1%)	2.81 (1.51:5.23)	< 0.001
Time up and go				
≤ 20	29 (18.7%)	126 (81.3%)	–	–
> 20	26 (89.7%)	3 (10.3%)	4.79 (3.37:6.80)	< 0.001^a^
Mini-mental (MMSE)				
≥ 23	50 (26.9%)	136 (73.1%)	–	–
< 23	48 (78.7%)	13 (21.3%)	2.93 (2.23:3.84)	< 0.001
Yesavage scale				
≤ 5	45 (25.0%)	135 (75.0%)	–	–
> 5	54 (78.3%)	15 (21.7%)	3.13 (2.36:4.15)	< 0.001
Gijón scale				
< 10	91 (38.1%)	148 (61.9%)	–	–
≥ 10	9 (75.0%)	3 (25.0%)	1.97 (1.37:2.84)	0.015^a^
Mini-nutritional				
> 23	11 (16.2%)	57 (83.8%)	–	–
17–23	59 (40.1%)	88 (59.9%)	2.48 (1.39:4.41)	< 0.001
< 17	28 (87.5%)	4 (12.5%)	5.41 (3.10:9.44)	< 0.001
Geriatric syndromes				
None	6 (7.1%)	79 (92.9%)	–	–
At least one	95 (56.6%)	73 (43.4%)	8.01 (3.66:17.52)	< 0.001
CIRS–G score				
0–1–2	20 (20.8%)	76 (79.2%)	–	–
> 2	66 (61.7%)	41 (38.3%)	2.96 (1.95:4.50)	< 0.001

^a^Fisher’s Exact Test

When variables were added to the model, some of those lost significance and negatively affected the strength of association of the other variables within the model. For example, the gait speed in the final model was excluded when the TUG was present, despite their lack of correlation. Clinically, when the TUG is abnormal, almost all variables are also affected in patients (26 out of 27). In contrast, an abnormal gait speed test does not necessarily mean that the TUG will be affected. It appears that the MNA effect gets diluted among the other variables, especially when it is included along with the Yesavage scale, MMSE, geriatric syndromes and TUG. This loss of effect could be due to the association of the MMSE with these variables. Additionally, the Gijon scale lost significance in the model, likely due to its low prevalence (only 12 of the patients included in this study had had an abnormal result).

In the bi-variate analysis, age > 85 years was associated with an abnormal CGA (67.7%) at a nearly twofold higher level than that of in the 65–74 range. Gender and level of education were not associated with frailty as defined by the formal CGA. All geriatric scales were associated with a diagnosis of frailty per the CGA criteria, except for the STOPP evaluation. Prior to the multivariate analysis, co-linearity was evaluated and a significant association was found between the Lawton and Barthel scales (*r*^2^ = 0.53), TUG and Katz index (*r*^2^ = 0.12) and the number of geriatric syndrome conditions and the CIRS-G score (*r*^2^ = 0.15). Therefore, the Barthel and Katz index, and the CIRS-G were not considered in the initial model.

### Multivariate Analysis

**Fig. 1:**
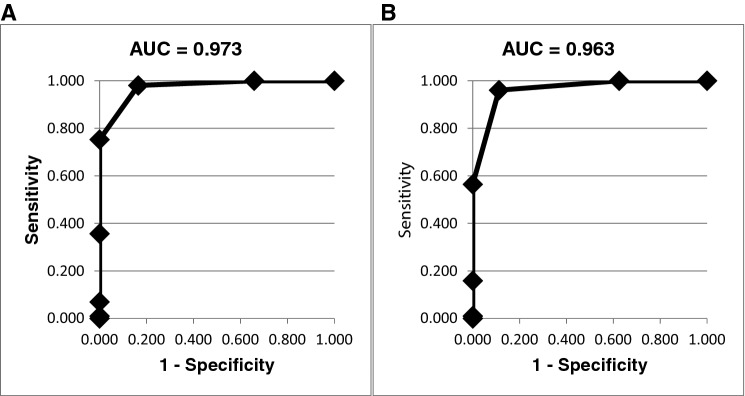
ROC curves of the multivariate model using the entire population. (A) ROC curve of the multivariate model using 6 variables. (B) ROC curve of the multivariate model using 5 variables

Our multivariate analysis resulted in 6 variables: polypharmacy; IADL; functional status, measured by the TUG; cognitive status using the MMSE; psychiatric status, measured by the Yesavage scale; and the presence of a geriatric syndrome, out of the 9 domains being significantly associated with frailty, as defined by the CGA. During the model creation, gait speed, Gijon scale and MNA were not found to be significant and were excluded from the model incorporating the aforementioned 6 variables. Results of the final age-adjusted model are shown in Table 3. The area under the curve (AUC) of the model is shown in Fig. 1. While we did not establish superiority of any tool or specific domain, these 6 domains were found to have the ability of assessing frailty and could be used instead of a complete, formal CGA, at least in our study population.

**Table 3:** Multivariate model of frailty adjusted by age and using 6 variables

Variables		Odds ratio ( 95% CI)		*p* value
Polypharmacy	> 5 drugs	773.1	(4.5:132,134.4)	0.011
Lawton scale	< 7	385.2	(20.1:7363.5)	< 0.001
Time Up and Go	> 20	124.6	(6.2:2489.8)	0.002
Mini-mental (MMSE)	< 23	53.5	(3.7:778.8)	0.004
Yesavage scale	> 5	118.7	(11.2:1254.5)	< 0.001
Geriatric syndromes	At least one	235.6	(13.3:4158.7)	< 0.001

Cases included: 183

Degrees of Freedom: 7

Log Likelihood Final: 45.9

Likelihood ratio: 177.8 (*p* < 0.001)

To further simplify the CGA, we proceeded to exclude those variables with the lowest strength of association from the final model. The latter consisted of 5 variables (excluding the MMSE) and yielded very similar results to the model comprised by six variables, with a slight increase in the log-likelihood and a slight decrease in the AUC to 0.963 (Fig. 1). When the MMSE was removed, the log likelihood increased from 45.9 to 58.1, and the model AUC decreased from 0.973 to 0.963 (Table 4 and Fig. 1). When a second variable (the Yesavage scale) was removed, the log likelihood increased from 58.1 to 88.4 and the AUC decreased to 0.925.

**Table 4:** Multivariate model of frailty adjusted by age and using 5 variables

Variables		Odds ratio (IC 95%)		*p* value
Polypharmacy	> 5 drugs	213.0	(5.0:9045.2)	0.005
Lawton scale	< 7	88.0	(12.1:642.5)	< 0.001
Time Up and Go	> 20	57.6	(5.2:643.7)	0.001
Yesavage scale	> 5	43.3	(8.8:213.0)	< 0.001
Geriatric syndromes	At least one	124.8	(11.4:1370.5)	< 0.001

Cases included: 183

Degrees of freedom: 6

Log Likelihood Final: 58.1

Likelihood ratio: 165.6 (*p* < 0.001)

## Discussion

This study included a moderate number of participants (253) and focused on the geriatric population with a diagnosis of NHL ranging in ages from 65 to 92 years. We believe that this study was able to discern the associations among the different geriatric evaluations to determine specific domains highly associated with frailty. These results could be potentially extrapolated to similar populations affected by other malignancies. In our cohort of patients, we found a high rate of frailty (evaluated by a formal CGA), with 40% of patients having ≥ 2 domains affected (Table 1). This prevalence lies within that reported by Tucci et al. in 2009, with only 50% of patients found to be “fit” for DLBCL treatment [20], and by Merli et al. in 2014 (where approximately 30% of patients were considered to be frail) [14]. Only 13.8% of our patients did not have any of the domains affected and, on average, each patient was found to have impairments in 2–3 domains. This underscores the fact that these patients had significant geriatric problems which would likely not have been detected by performance status tools commonly used in oncology practice (e.g. ECOG or KPS). It would have been of interest to compare these results with those populations with other malignancies, or in the non-cancer geriatric populations, to determine whether this prevalence of frailty is inherent to the Peruvian geriatric population, or observed in higher proportions among the older NHL patients.

There was high variability among the different components of the CGA. It is noteworthy that in our patient population, polypharmacy and meeting STOPP criteria were not as prevalent as compared to other studies, which showed higher frequency of these variables, as anticipated, given that polypharmacy is a common geriatric problem. It is possible that because this was a government institution, the rationing or administration of medications may be stricter, as compared to private or academic hospitals. The access and availability of medications, even when they are indicated and prescribed, pose a known challenge in government-run hospitals which, in addition to rationing practices, could impact the number of medications that these patients are on. With respect to the measurement of ADLs, it was noteworthy that different scales could show different results, despite being similar in what they captured. We postulate that these differences could be due to the sensitivity of the tests, and dependent on the operator performing these tests. In our study, the Yesavage scale disclosed higher rates of depression than reported by others [17]. The MMSA in our cohort found cognitive impairment to be similar to that from a previous study in cancer patients [18]. Of interest, because the patients seen in our government-run hospital were typically retirees on a state pension, it is not surprising that the Gijon scale score evaluating social support and economic situation was low. Two-thirds of our patients had at least one geriatric syndrome (excluding dementia, delirium, depression and polypharmacy); this is a higher incidence of geriatric syndromes and comorbidities in our population compared to that reported in an Italian study [19]. The presence of a geriatric syndrome had the strongest association with frailty, however. Similarly, over half of the subjects had a CIRS-G score ≥ 2, reflecting the high prevalence of comorbidities.

Our multivariate analysis resulted in 6 (polypharmacy; IADL; functional status, measured by the TUG; cognitive status using the MMSE; psychiatric status, measured by the Yesavage scale; and the presence of a geriatric syndrome) out of the 9 domains as being significantly associated with frailty, as defined by the CGA and, hence, related to negative clinical outcomes, which is also described elsewhere [6, 7, 13]. While we did not establish superiority of any tool or specific domain, at least in our study population, these 6 domains were found to have the ability of assessing frailty and could be used instead of a complete, formal CGA. Recently, Merli et al. provided data to support the use of a simplified CGA for the initial evaluation of older patients with DLBCL, and incorporated their simplified CGA to build a new, validated prognostic score, the Elderly Prognostic Index (EPI) to ultimately predict overall survival [21].

A main weakness of our analysis is that it was unable to predict for clinical outcomes such as treatment tolerance, compliance, adverse effects or mortality. While that was not the aim of this study, comprehensive geriatric assessments have been shown to predict outcomes more accurately, compared to ECOG PS or KPS [22, 23]. Another significant limitation of this study is the lack of a validation set, and a confirmatory study in a comparable population, perhaps another government facility in Peru or in Latin America at large, would be ideal in addressing this limitation. Our future plan is to study CGAs within the Consenso del Grupo de Estudio Latinoamericano de Linfoproliferativos (GELL). An additional limitation of this study is that it was performed in a Peruvian population at a government hospital and, thus, generalizability would be a concerning point. Future studies within the GELL and involving multiple institutions would be able to address the latter issue.

## Conclusions

We conclude that a geriatric evaluation based on only 5 measures (polypharmacy, Lawton scale, TUG, Yesavage scale and the presence of a geriatric syndrome) performed similarly in predicting frailty, when compared to a formal CGA in our patient population of older adults with a diagnosis of NHL. Future studies will be needed to determine whether the selective domains identified in this study could be of utility in creating predictive and prognostic indices potentially applicable to other malignancies and other populations.
